# Calcium depletion destabilises kinetochore fibres by the removal of CENP-F from the kinetochore

**DOI:** 10.1038/s41598-017-07777-6

**Published:** 2017-08-04

**Authors:** Rinyaporn Phengchat, Hideaki Takata, Susumu Uchiyama, Kiichi Fukui

**Affiliations:** 1 Graduate School of Engineering, Osaka University, 2-1 Yamadaoka, Suita 565-0871 Osaka, Japan; 2 Biomedical Research Institute, National Institute of Advanced Industrial Science and Technology (AIST), 1-8-31, Midorigaoka, Ikeda 563-8577 Osaka, Japan; 3 Chromosome Engineering Research Centre, Tottori University, 86 Nishimachi, Yonago 683-0826 Tottori, Japan

## Abstract

The attachment of spindle fibres to the kinetochore is an important process that ensures successful completion of the cell division. The Ca^2+^ concentration increases during the mitotic phase and contributes microtubule stability. However, its role in the spindle organisation in mitotic cells remains controversial. Here, we investigated the role of Ca^2+^ on kinetochore fibres in living cells. We found that depletion of Ca^2+^ during mitosis reduced kinetochore fibre stability. Reduction of kinetochore fibre stability was not due to direct inhibition of microtubule polymerisation by Ca^2+^-depletion but due to elimination of one dynamic component of kinetochore, CENP-F from the kinetochore. This compromised the attachment of kinetochore fibres to the kinetochore which possibly causes mitotic defects induced by the depletion of Ca^2+^.

## Introduction

During the mitosis of animal cells and some fungi, spindle microtubules grow from the microtubule-organising centre to capture and align chromosomes at the cell equator. Microtubules attach to the kinetochore located at the chromosome centromere and these microtubules are called kinetochore microtubules (or K-fibres)^1^. The attachment of microtubules to kinetochores of each sister chromatid is crucial for successful cell division. Efficient kinetochore-microtubule attachment is determined by the assembly of intact kinetochore and microtubule dynamics, including de-/polymerisation. Defects in these components impair attachment and chromosome alignment at the spindle equator, which possibly leads to mitotic arrest.

Ca^2+^ is a universal secondary messenger involved in several processes inside cells. Concentration of Ca^2+^ in cells is critical for the cell cycle progression. During mitosis, intracellular Ca^2+^ increases and induces chromosome condensation, spindle fibre formation and chromosome segregation, which are processes required for successful cell division^2–4^. The precise role of Ca^2+^ on spindle fibre formation during mitosis remains unresolved, although several previous studies imply their relationship. According to Xu *et al*., the mitotic spindle structure is affected by Ca^2+^-depletion in the concentration dependent manner, in which the mitotic spindle continues to shrink under the treatment, suggesting microtubule disassembly^5^. An increase in the Ca^2+^ concentration during the mitosis is thought to induce microtubule depolymerisation leading to anaphase onset^4^, however the injection of Ca^2+^ saturated calmodulin during prometaphase prolongs the duration to anaphase onset^3^ similar to the injection of EGTA-CaCl_2_ solution to lower intracellular Ca^2+^ concentration beneath 100 nM^2^, and the shortened spindle microtubules was observed in both treatments. Factors required for microtubule polymerisation have been characterised using *in vitro* systems and it was found that self-assembly of microtubules from brain tubulin extracts requires the presence of Mg^2+^ and GTP^6–8^, and Ca^2+^ should be removed using chelating reagents^9^. However this might not fully explain the effect of Ca^2+^ on microtubule polymerisation in a living cell which is more complicated. According to Ochieng *et al*., human breast epithelial cells displayed extensively polymerised cytoplasmic microtubule complexes when cultured in high Ca^2+^ medium. While in low Ca^2+^ medium, tubulins in majority of the cells were in depolymerised state^10^. This suggests that high level of Ca^2+^ favours either microtubule polymerization or stabilises the polymerised state of tubulin.

Depletion of intracellular Ca^2+^ during mitosis also causes global chromatin decondensation^11^. Because the assembly of kinetochore occurs in stepwise manner from the innermost to outermost layer, decondensed chromatin might interfere the formation of heterochromatin which makes up centromeric chromatin that supports kinetochore structure^12, 13^, or interrupting the organisation of the constitutive centromere associated network (CCAN) by affecting centromeric chromatin folding similar to CENP-depleted chromosome^14^.

In this study, we present an additional role of Ca^2+^ during mitosis, particularly the effects of Ca^2+^-depletion on spindle fibre formation and stability in the cells. We found that the depletion of Ca^2+^ during mitosis reduced the stability of kinetochore microtubules. The effects of Ca^2+^-depletion on spindle fibre formation were confirmed for the first time, with live cell imaging showing that the formation of spindle microtubules was not inhibited by the depletion of Ca^2+^. The loss of spindle fibre stability under Ca^2+^-depletion was the most likely due to the loss of CENP-F (mitosin), a dynamic component of the fibrous corona, from the kinetochores.

## Results

### Microtubule Stability after Ca^2+^-Depletion

**Figure 1:**
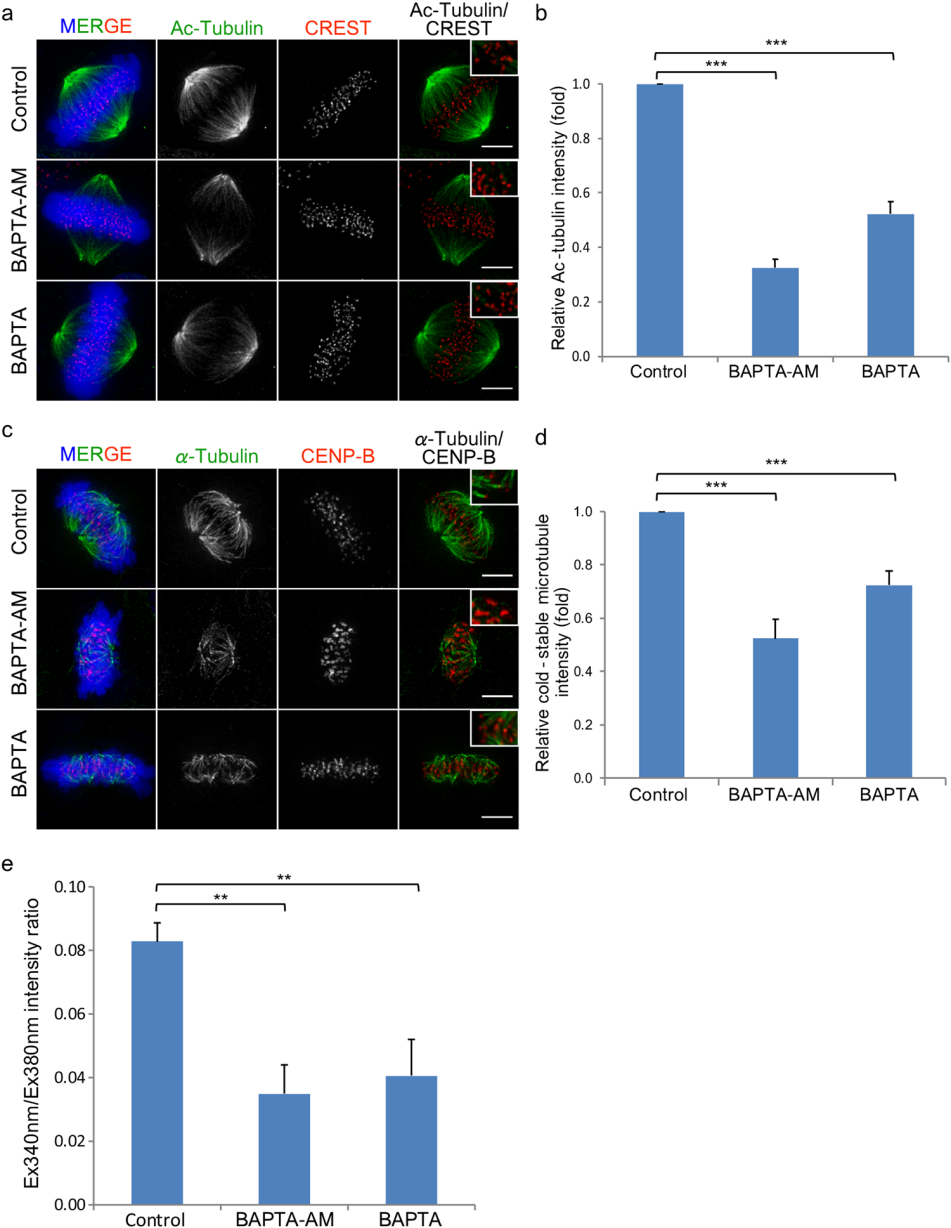
Ca^2+^-depletion reduces the stability of spindle fibres. (**a**) HeLa^WT^ cells arrested at metaphase using MG132 were treated with 25 µM BAPTA-AM or 10 mM BAPTA and 5 µM ionomycin to reduce intracellular Ca^2+^ then stained with anti-Ac-tubulin antibody. (**b**) A graph presenting the relative Ac-tubulin intensity at kinetochore of each treatment. (**c**) Fluorescence images of metaphase cells in control, BAPTA-AM and BAPTA (with ionomycin) treatments after exposure to low temperature. (**d**) A graph presenting the relative cold-stable microtubule intensity at kinetochore of each treatment. (**e**) A bar graph showing the intensity ratio of Fura-2 excited at 340 and 380 nm measured in mitotic cells, which represent intracellular calcium levels. Error bars indicate standard deviations derived from three independent experiments. Bar, 5 µm.

Initially, to investigate the effects on the depletion of intracellular Ca^2+^ on the stability of kinetochore fibre, microtubules with acetylated tubulin (Ac-tubulin) which have slower turnover rate than non-acetylated microtubules, indicating higher stability^15^, was detected. HeLa^WT^ cells were arrested at metaphase using MG132, a proteasome inhibitor, without disruption to spindle fibres, and immunofluorescence assay against Ac-tubulin was performed. In control metaphase cells, intense signal of Ac-tubulin was observed through the length of kinetochore fibres, showing stable microtubules (Fig. 1a). Upon the treatment with either BAPTA-AM or BAPTA and ionomycin, Ac-tubulin signals near CREST signals were diffused especially in BAPTA-AM treated cells. Relative Ac-tubulin intensity showed more than two-fold reduction of Ac-tubulin at kinetochore in BAPTA-AM treatment (Fig. 1b). A significant decrease of Fura-2 Ex340 nm/Ex380 nm intensity ration was observed in both conditions, confirming the reduction of intracellular Ca^2+^ level (Fig. 1e).

In addition to the acetylation of tubulin, the stability of kinetochore fibres was also examined by cold treatment. When both ends of microtubules stably attach, they do not disassemble at low temperature^16, 17^. Subsequent to BAPTA-AM or BAPTA and ionomycin treatment, cells were exposed to ice-cold medium to remove unstable spindle fibres, which was the same methodology used in the previous report^18^. Microtubule stability was evaluated by measuring the intensity of tubulin signals located adjacent to kinetochores. Both the Ca^2+^-depletion treatments abolished cold-stable microtubules (Fig. 1c) and significantly reduced the relative cold-stable microtubule intensity (Fig. 1d), indicating less microtubules attached to kinetochores. However, the effect of BAPTA-AM on the microtubule stability was more severe than BAPTA and ionomycin treatment because BAPTA-AM treatment caused the two-fold reduction of the cold-stable microtubule index when compared with that of BAPTA and ionomycin (Fig. 1d). Both Ac-tubulin and cold-stable microtubule indicate the reduction of kinetochore fibre stability, implying that the attachment of kinetochores and spindle fibres was unstable in the Ca^2+^-depleted cells.

### Effect of Ca^2+^-Depletion on Microtubule Polymerisation

Depletion of Ca^2+^ may directly affect de-/polymerisation of spindle fibres, resulting in the reduction of cold-stable kinetochore microtubules. The dynamics of microtubule polymerisation during Ca^2+^-depletion were observed using time-lapse observation of U2OS cells expressing CENP-A-GFP and mCherry-α-tubulin. Metaphase U2OS cells were briefly treated with nocodazole to remove the spindle fibres and the cells were then allowed to repolymerise the microtubules by release of the cells into the fresh medium with or without Ca^2+^-chelating drugs.

**Figure 2:**
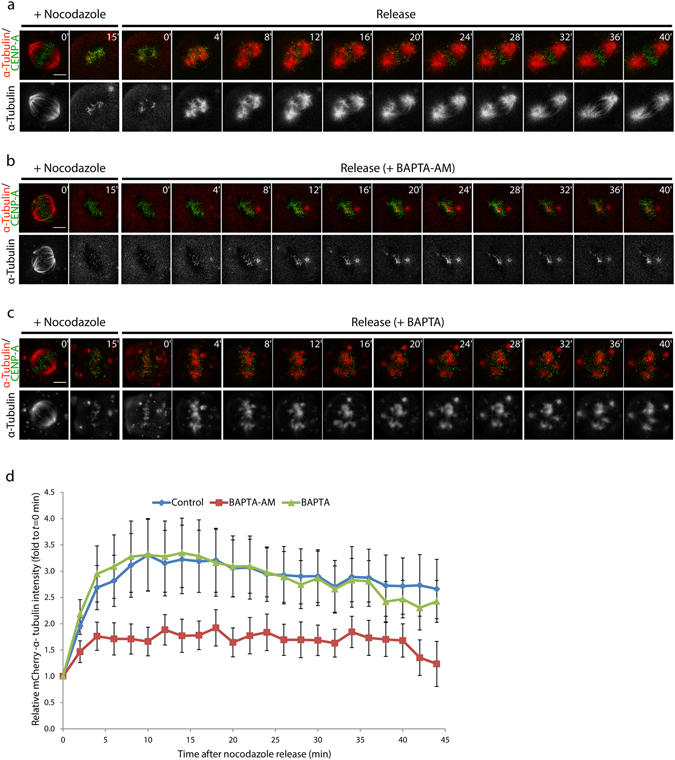
BAPTA-AM treatment inhibits microtubule re-polymerisation. (**a**–**c**) Time-lapse images of U2OS cells expressing CENP-A-GFP and mCherry-α-tubulin during microtubule de-/re-polymerisation assay. Cells were released from nocodazole into fresh medium containing drugs as indicated. (**d**) A graph presenting the relative mCherry-α-tubulin at kinetochore of each treatment. Error bars indicate standard errors (*n = *15 cells for control, *n = *12 cells for BAPTA-AM, and *n = *8 cells for BAPTA). Bar, 5 µm.

Before treatment of the cells with nocodazole, metaphase cells with intact spindle fibres were observed and chromosomes were confirmed to be all aligned at the metaphase plate (Fig. 2). After 10–15 min of nocodazole treatment, the organisation of spindle fibres was completely destroyed. Weak mCherry-α-tubulin signals were detected throughout the cytoplasmic area. In some cells, chromosomes were dispersed in the cytoplasm and no longer aligned at the metaphase plate. Conversely, some cells showed that chromosomes maintained their alignment at the metaphase plate.

In the control, repolymerisation of microtubules was observed within 5 min after release from nocodazole and continued until ~30 min after the release (Fig. 2a). Re-arrangement of chromosomes was also observed. After 1 h release from nocodazole, chromosomes were aligned at the metaphase plate, although the alignment was not similar to that before nocodazole treatment.

When arrested cells were released from nocodazole into medium containing BAPTA-AM, repolymerisation of a small amount of mCherry-α-tubulin was observed during the first 20 min (Fig. 2b). However, microtubules did not further repolymerise, and 1 h after nocodazole release, re-organisation of spindle fibres was not observed. The cells were also released into the medium containing BAPTA and ionomycin, which could deplete Ca^2+^ inside the cells similar to BAPTA-AM (Fig. 2c). In contrast to BAPTA-AM treatment, repolymerisation of microtubules was obviously observed shortly after release from nocodazole, showing as an accumulation of plenty of mCherry-α-tubulin on chromosome area. Similar to control, the microtubule repolymerisation gradually continued until ~30 min after nocodazole release coupling with chromosome re-arrangement. However, the alignment of chromosomes at metaphase plate could not be observed at 1 h after nocodazole release.

Total mCherry-α-tubulin intensity at each time point after the release of nocodazole was measured and compared with the intensity at the first time point after release (*t = *0 min, Fig. 2d). In the control, total mCherry-α-tubulin intensity increased 3-fold (relative to *t = *0 min) within 10 min after the release from nocodazole treatment and remained above 2-fold increase until the end of the observation. A similar phenomenon was observed in the treatment with BAPTA and ionomycin, in which 3-fold increase in total mCherry-α-tubulin intensity was also observed within 10 min after the release of nocodazole in BAPTA-treated cells. In BAPTA-AM treatment, although the total mCherry-α-tubulin intensity increased after the release of nocodazole, the increment was obviously small compared with cells in control and BAPTA-ionomycin treatments. These results clearly indicate that Ca^2+^-depletion alone does not prevent microtubule polymerisation and the inhibition of microtubule repolymerisation by BAPTA-AM might be independent from its Ca^2+^-chelation activity (see Discussion).

### Localisation of Centromeric and Kinetochore Proteins under Ca^2+^-Depletion

**Figure 3:**
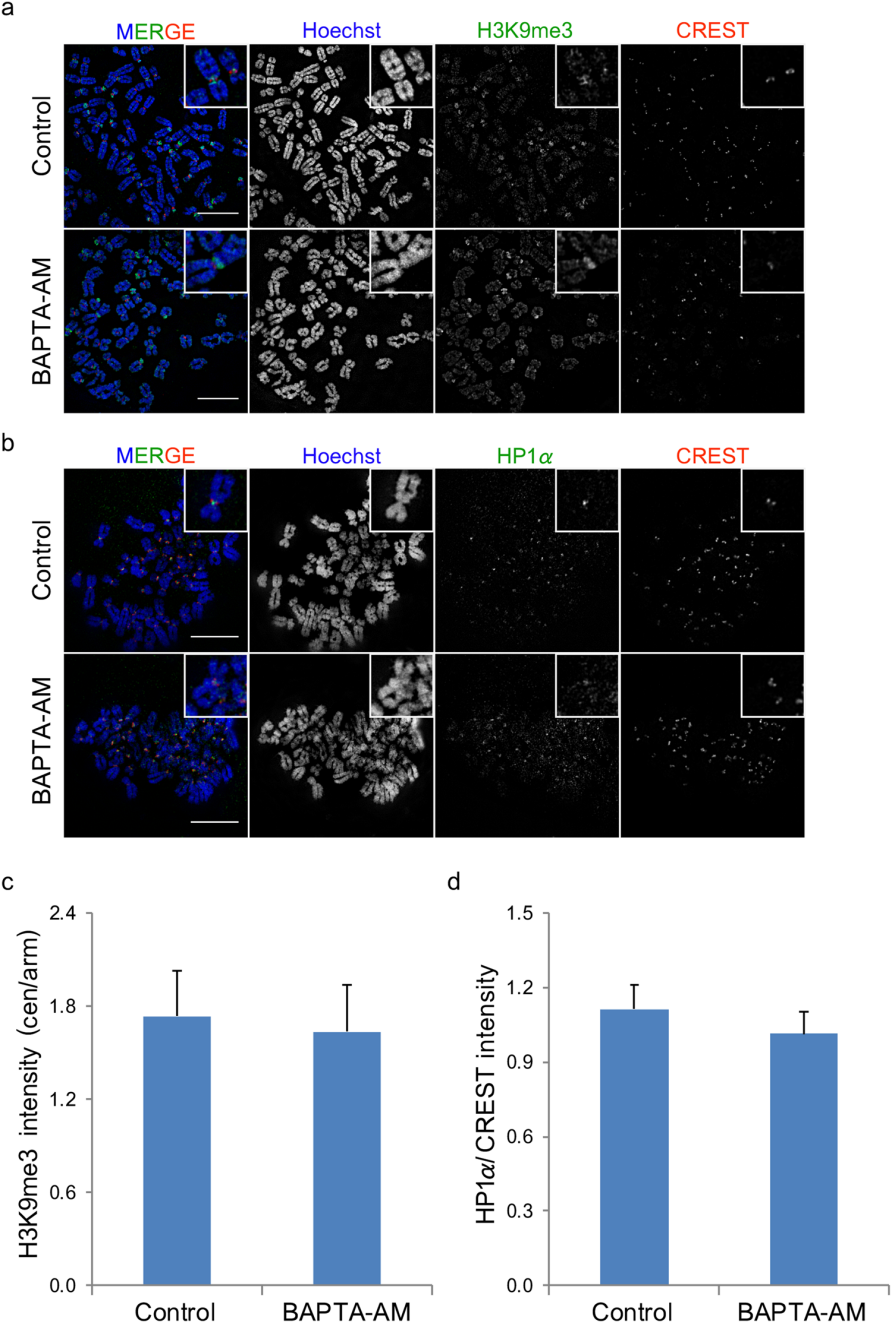
Ca^2+^-depletion does not alter H3K9me3 and HP1α localisation. Fluorescence images of chromosome spreads prepared from control and BAPTA-AM treated cells for the localisation of H3K9me3 (**a**) and HP1α (**b**). Bar graphs below indicate H3K9me3 centromere-to-arm signal intensity (**c**) and HP1α/CREST signal intensity (**d**). Error bars indicate standard deviations derived from three independent experiments. Bar, 5 µm.

The reduction of kinetochore fibre stability when Ca^2+^ was depleted might be due to defects in kinetochore structure because Ca^2+^-depletion induces chromosome decondensation^11^ and this possibly compromises the formation of kinetochore locating at centromere. Therefore the localisation of important centromeric proteins after Ca^2+^-depletion was investigated to understand the mechanism of how Ca^2+^-depletion induces unstable microtubule attachment to the kinetochore. Heterochromatin protein 1α (HP1α) localises at the centromere by recognising and binding to trimethylated histone H3 lysine 9 (H3K9me3), and provides itself as a mark to anchor other inner kinetochore proteins^19^. Mislocalisation of H3K9me3 due to chromosome decondensation may prevent the localisation of other centromeric proteins, including HP1α, to the centromere^18, 20^. H3K9me3 signals were detected at pericentromeric regions (and also some telomeric regions) of chromosomes, consistent with previous studies (Fig. 3a)^18–20^. Moreover, there was no significant difference in the centromere-to-arm H3K9me3 intensity ratio between control and Ca^2+^-depletion (Fig. 3c). This indicates that H3K9me3 localises normally to centromeric regions even though chromosomes are decondensed.

The localisation of HP1α at the centromere under the control and Ca^2+^-depletion conditions was examined. HP1α signals were detected in the middle position between the two CREST signals of all chromosomes in both intact cells and chromosome spreads, as shown in Fig. 3b. HP1α/CREST intensity ratios in Ca^2+^-depleted cells was not significantly different from that of control samples (Fig. 3d), indicating that HP1α could localise to the centromere normally even under the Ca^2+^-depletion condition.

**Figure 4:**
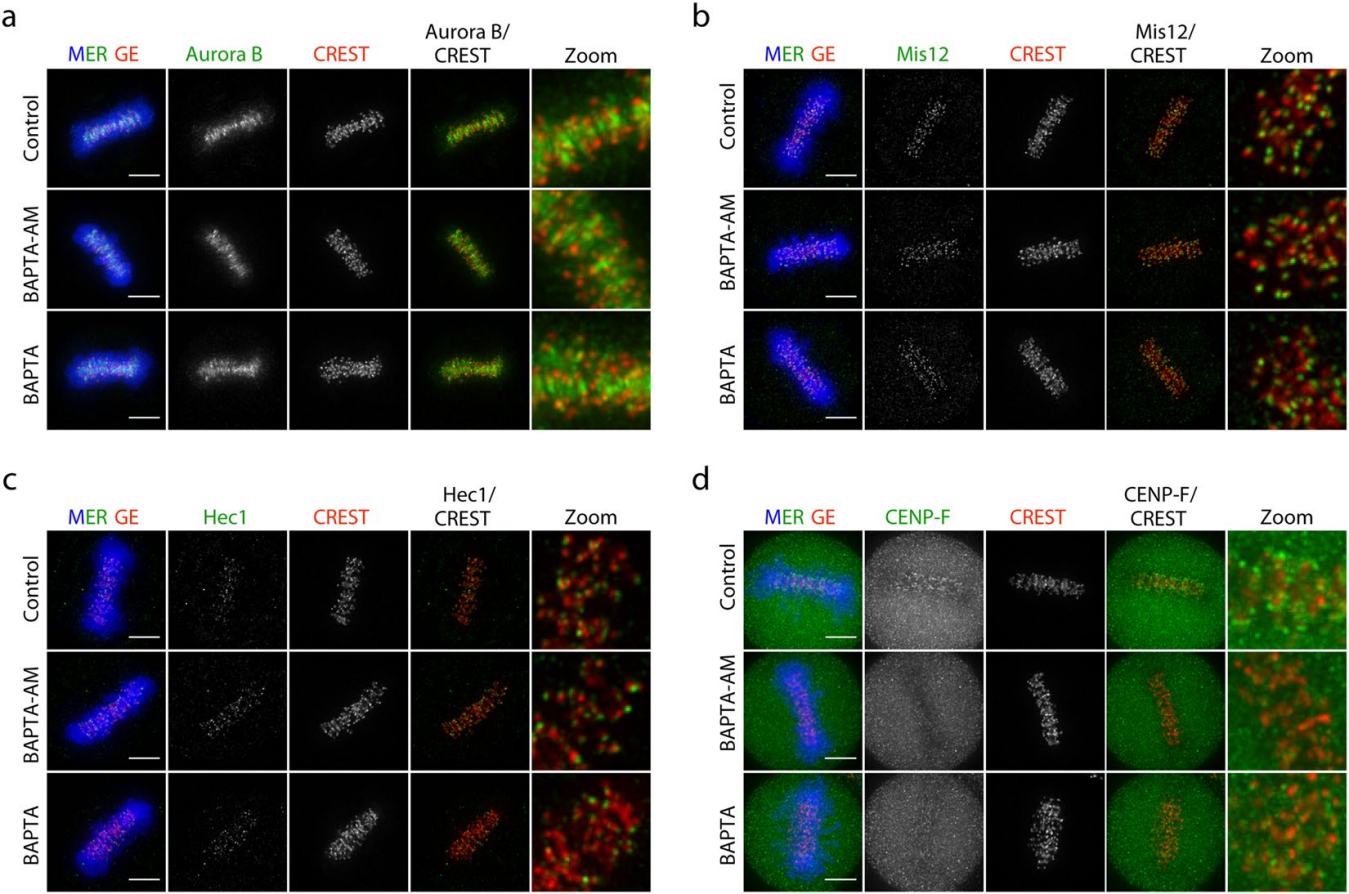
Ca^2+^-depletion causes defects in the localisation of CENP-F at the outer kinetochore. Fluorescence images of metaphase cells after control, BAPTA-AM and BAPTA (with ionomycin) treatments for the localisation of Aurora B (**a**), Mis12 (**b**), Hec1 (**c**) and CENP-F (**d**). Bar, 5 µm.

Localisation of several kinetochore proteins was examined. We first probed Aurora B, which is required for the interaction between inner and outer plates of kinetochore^21^ and found that in both the control and Ca^2+^-depleted cells, Aurora B localised normally to the centromere (Fig. 4a). Mis12, the outer plate kinetochore protein that interacts with HP1α for its localisation^22^, could be recruited to its authentic position even in the Ca^2+^-depleted cells (Fig. 4b). Hec1, another outer plate constitutive protein that locates to the microtubule–kinetochore interface^12^, also remained on the kinetochore when Ca^2+^ was depleted (Fig. 4c). Interestingly, a transient kinetochore component, CENP-F which locates distal to the outer kinetochore region was partially detected in the Ca^2+^-depleted cells by BAPTA-ionomycin treatments, and barely detectable in the BAPTA-AM treatment, while in the control cells, intense signals of CENP-F could be detected on the kinetochore (Fig. 4d). These results indicate that even though chromosomes were decondensed owing to Ca^2+^-depletion, both chromosome centromere and kinetochore, except the dynamic components such as CENP-F, remained intact, and loss of the dynamic kinetochore component results in a reduction of spindle fibre stability under Ca^2+^-depleted conditions.

## Discussion

In this study, we revealed that depletion of intracellular Ca^2+^ during mitosis reduced the stability of kinetochore microtubules which implied the unstable attachment between spindle fibres and the kinetochore. There are two possibilities explaining the reduction of stable spindle fibres upon Ca^2+^ depletion: (i) direct effects of Ca^2+^-depletion on the de-/polymerisation of microtubules and (ii) defects in kinetochore structure. For the first possibility, Ca^2+^-depletion by treatment of the cells with BAPTA-AM, BAPTA-AM derivatives or EGTA may inhibit microtubule polymerisation because these treatments decrease the total amount of ATP inside cells^23^, and in the presence of GTP, ATP increased the rate and extent of microtubule assembly^24^. However, as demonstrated by live cell imaging, Ca^2+^-depletion did not interfere with the microtubule polymerisation process (Fig. 2c). Only BAPTA-AM could inhibit the polymerisation of microtubules, and this property was independent of its Ca^2+^-chelation activity. This is consistent with previous reports using *in vitro* systems showing that the depletion of Ca^2+^ using EGTA allowed the polymerisation of rat brain tubulin in the presence of Mg^2+^ together with either ATP or GTP^9^, and in rat fibroblasts^23^ and adipocytes^25^. The inhibition of microtubule polymerisation might be caused by the aromatic rings of BAPTA, because the treatment with either BAPTA-AM or D-BAPTA-AM, which reduces Ca^2+^-chelating capacity, leads to microtubule depolymerisation in RAT2 cells^23^. Therefore, depletion of Ca^2+^ seems to have no direct effect on the polymerisation of microtubules.

For the second possibility, kinetochore structures might be impaired owing to Ca^2+^ depletion because Ca^2+^-depletion during mitosis induced chromosome decondensation^11^, and this may compromise kinetochore-microtubule attachment. Several proteins including kinetochore components and those important for kinetochore assembly were probed. The position of H3K9me3, which serves as a mark for the binding of other centric proteins, was examined. Centromeric chromatin contains H3K9me3, which is a binding site of HP1. Loss of HP1 from the centromere affects the binding of other centromeric proteins, resulting in abnormal microtubule attachment. As a result, this leads to chromosomal instability^18^. H3K9me3 and SUV39H1, which is a histone methyltransferase (HMTase) responsible for H3K9me3 but not H3K9me1 and H3K9me2, increases in mitotic cells^26^. H3K9me3 is lost from the centromeric regions of the cells with chromosome instability. Reduction of H3K9me3 and HP1 from chromatin centromeric regions was detected after TSA treatment, which caused global chromatin decompaction in HTC116 nuclei^20^. Pericentromeric localisation of H3K9me3 could be regulated by altering SUV39H1 activity. Disruption of the *SUV39H1* gene led to the loss of H3K9 methylation from pericentric heterochromatin. SUV39H1 activity is inhibited by the binding of deleted in breast cancer 1 (DBC1) to its catalytic domain, preventing SUV39H1 methylating histone H3. Factors, for example, tumour suppresser gene, *KLLN*, can also regulate H3K9me3 by altering SUV39H1 activity through interaction with DBC1^27^. Interestingly, the C-terminus of DBC1 contains an EF hand domain that binds Ca^2+^. However, this EF hands may not be functional because it is unlikely to bind to Ca^2+^, as observed for the Nudix domain^28^. In addition, Ca^2+^ contributes to chromosome condensation after NEB, unlike other chromosome condensation factors, such as Topoisomerase IIα and histone post translational modifications. H3K9 may already be methylated by SUV39H1 and localise to the centromere before Ca^2+^ gains access to chromosomes to promote condensation. Therefore, the localisation of H3K9me3 in less compact chromosomes-induced by Ca^2+^-depletion remained unchanged, and the localisation of HP1, which recognises and binds to H3K9me3, was not affected by Ca^2+^-depletion.

Similar to H3K9me3 and HP1α, the localisation of Aurora B kinase to the centromere was not affected by Ca^2+^-depletion. Some conserved kinetochore components, such as Ndc80, KNL1 and Dsn1, which are crucial for kinetochore assembly, require Aurora B for their localisation^29, 30^. Since Aurora B remained at the centromere under the condition of Ca^2+^-depletion, the localisation of those kinetochore proteins should not be impaired.

Depletion of Ca^2+^ also did not prevent the localisation of static components of kinetochore, Mis12 and Hec1, but affected kinetochore localisation of CENP-F. Mis12 directly interacts with HP1α^31^ and the localisation of Mis12 to the outer kinetochore requires HP1α, because a reduction of HP1α by RNAi abolishes kinetochore localisation of Mis12^32^. Because of the observation that HP1α could localise normally to the centromere when Ca^2+^ was depleted, the recruitment and localisation of Mis12 to the kinetochore was not disrupted. Hec1 is another static outer kinetochore component that directly interacts with spindle fibres at the kinetochore-microtubule interface. Hec1 requires Aurora B phosphorylation at its N-terminus to regulate microtubule attachment and plus-end microtubule polymerisation^33^. Loss of Hec1 from the kinetochore results in complete loss of kinetochore-microtubule attachment^34^. However, when Ca^2+^ was depleted, Aurora B localised normally to the centromere and some microtubules remained attached to the kinetochore. Thus, the localisation and function of Hec1 should not be affected by the depletion of Ca^2+^.

In addition to those immobilised components, there are several proteins that transiently localise at the distal region of the outer kinetochore. These proteins also facilitate the interaction between kinetochore and the plus-end of microtubules, and de-/stabilise microtubules. In this study, we found that CENP-F was abolished when Ca^2+^ was depleted. CENP-F is essential for the firm assembly of the kinetochore-microtubule interface because CENP-F depleted cells exhibited mitotic delay and a decrease in the stability of kinetochore microtubules^35–38^. Although the interaction between CENP-F and microtubules, and its importance for microtubule–kinetochore attachment are well studied, the recruitment of this protein to the kinetochore is still inconclusive. A spindle checkpoint protein Bub1 and a kinetochore scaffold KNL1 were reported to be crucial for localisation of CENP-F to the kinetochore^39, 40^. The recruitment of CENP-F to kinetochore could also be mediated by a small GTPase Rab5^41^. Rab5-silenced cells exhibited mitotic delay and chromosome misalignment, similar to CENP-F-depleted cells^35, 37, 41^. Rab5 formed a complex with CENP-F, and promoted efficient localisation of CENP-F to kinetochore^41^. Besides Rab5, the contribution of Rab11, the other Rab family protein, to the organisation of mitotic spindle was also reported^42^. Depletion of Ca^2+^ might inhibit small GTPase activity either through guanine nucleotide exchange factors possessing EF hands, or the binding of Ca^2+^/calmodulin to the small GTPases^43^. Severe reduction of the tubulin acetylation and cold-stable microtubule in BAPTA-AM treatment (Fig. 1b,e) might be caused by a combination of the loss of CENP-F from the kinetochore and the direct inhibition of microtubule polymerisation by BAPTA-AM, as mentioned above.

In conclusion, Ca^2+^-depletion has no direct effects on the de-/polymerisation of microtubules during mitosis. However, it is required for the localisation of some transient components located at the kinetochore-microtubule interface that are important for the establishment of intact kinetochore-microtubule attachment. Future study to assess the localisation of other dynamic components of the kinetochore beside CENP-F and to clarify how Ca^2+^ can regulate the localisation of these proteins will be anticipated.

## Materials and Methods

### Cell Culture and Synchronisation

HeLa^WT^ cells and U2OS cells expressing GFP-tagged CENP-A and mCherry-tagged α-tubulin were cultured in Dulbecco’s Modified Eagle Medium (DMEM; GIBCO BRL) supplemented with 10% fetal bovine serum (Biosera) at 37 °C with 5% CO_2_. The following drugs were used for cell treatments: thymidine (2.5 mM; Sigma), nocodazole (80 ng/ml; Sigma), MG132 (10 µM: Calbiochem), BAPTA-AM (25 µM; Dojindo), BAPTA (10 mM; Dojindo), ionomycin (5 µM; Calbiochem) and Fura-2 am (1 µM; Dojindo).

### Antibodies

The following primary antibodies were used for indirect immunostaining: mouse monoclonal anti-α-tubulin (1:1,000; Calbiochem), mouse monoclonal anti-acetylated tubulin (1:1,000; Sigma-aldrich), rabbit polyclonal anti-CENP-B (1:1,000; Millipore), rabbit polyclonal anti-histone H3K9me3 (1:2,500; Millipore), mouse monoclonal anti-HP1α (1:400; Millipore), rabbit polyclonal anti-Aurora B (1:500; Abcam), mouse monoclonal anti-HEC1 (1:200; Abcam), rabbit polyclonal anti-Mis12 (1:200; Abcam), mouse monoclonal anti-CENP-F (1:200; Abcam), and human anti-centromere autoantibody (CREST, 1:1,000; Cortex Biochem).

### Intracellular Ca^2+^ Measurement

Levels of intracellular Ca^2+^ was measured using Fura-2 am as previously described in Phengchat *et al*.^11^. In brief, before the treatment with BAPTA-AM or BAPTA and ionomycin, cells were incubated with Fura-2 am for 45 min at room temperature and further 20 min at 37 °C to allow completely hydrolysis of AM ester. Medium was replaced with fresh phenol red-free medium containing BAPTA-AM or BAPTA and ionomycin. After 2 h incubation, the cells were placed on of the inverted microscope equipped with a temperature-controlled chamber set at 37 °C with CO_2_ supply (Olympus IX71) and a 40× 1.35 UApo/340 Iris oil immersion objective lens. Fluorescence images of Fura-2 were sequentially acquired by 340 and 380 nm excitation wavelength and measured emission at 510 nm. Quantification of fluorescence at 510 nm after excitation at 340 and 380 nm (F340/F380 ratio) was used as an index for intracellular Ca^2+^ levels using ImageJ (National Institute of Health).

### Immunofluorescence

HeLa cells were cultured on glass coverslips and synchronised with double thymidine block. Cells were arrested at metaphase using MG132 for 1 h then treated with either BAPTA-AM or BAPTA and ionomycin for 2 h. For microtubule stability (cold-treatment), all coverslips were transferred to ice-cold medium and incubated for 20 min on ice before fixation. For choromosome spreads, HeLa cells were synchronised with double thymidine blocks and arrested in prometaphase with nocodazole for 12 h then treated with BAPTA-AM for 2 h. Cells were collected for the metaphase-chromosome spread using the following method. Cells were treated with hypotonic solution (75 mM KCl) for 15 min at 37 °C and were cytospun onto coverslips. Cells were fixed with either 4% para-formaldehyde for 10 min and permeabilised using 0.5% Triton X-100/PBS for 10 min, or ice-cold methanol at −20 °C for 10 min. Cells were blocked with 3% BSA/PBS for 1 h. All primary antibody reactions were performed at 4 °C overnight, except for HP1α, which was performed at 37 °C for 20 min. Secondary antibody reactions were performed at room temperature for 1 h. DNA was counterstained with Hoechst 33342. Samples were mounted in Vectorshield mounting medium (Vector Laboratories).

Images were taken with a DeltaVision deconvolution microscope (IX71, Olympus, Tokyo, Japan) using a 1.4 NA PlanApo 100× oil immersion objective (Olympus). The z-stack distance was 0.5–1 µm and the total z-stack thickness was 15–18 µm. Raw 3D images were deconvoluted using constrained iterative deconvolution and converted to 2D images with maximum projection using softWoRx (AppliedPrecision). Images were processed by background subtraction and fluorescence intensities were individually scaled to 0–255. Image processing and intensity measurements were performed using ImageJ.

### Time-Lapse Observations

U2OS cells cultured in 35 mm poly-L-lysine coated glass bottom dishes were synchronised with double thymidine blocking and DNA was stained with Hoechst 33342. Before performing time-lapse observations, the medium was discarded and replaced with phenol red-free DMEM containing 10% fetal bovine serum, 4 mM L-glutamine and 10 mM HEPES for microscopic observations. The cells were monitored under the temperature-controlled stage of the DeltaVision deconvolution microscope equipped with a CO_2_-chamber set at 37 °C with a 100× 1.4 NA PlanApo 100× oil immersion objective (Olympus). For the microtubule depolymerisation/repolymerisation assay, metaphase cells with intense mCherry-α-tubulin and CENP-A-GFP signals were chosen. The cells were treated with 0.5 or 1 µg/ml nocodazole for 10–15 min, washed with PBS three times then released into pre-warmed fresh medium (containing indicated drugs) to allow microtubule repolymerisation. The z-stack images were taken every 2 min during release. Images were processed by background subtraction and fluorescence intensities were individually scaled to 0–255. Spindle microtubule regions were specified by segmented the grey scale images of mCherry-α-tubulin with auto-threshold function. Relative mCherry-α-tubulin intensity in spindle microtubule regions was calculated by normalising total fluorescence intensity of mCherry-α-tubulin at each time point after the release of nocodazole with that of the first time point (*t = *0 min).

### Statistical Analysis

Statistical analysis was carried out in R using the Mann-Whitney-Wilcoxon test to compare the mean values. ‘*’, ‘**’ and ‘***’ indicate *p* < 0.05, 0.01 and 0.001, respectively.
